# Pseudomonas aeruginosa infection is associated with alveolar macrophage M2 polarization via MERTK-mediated NLRC4 inflammasome activation

**DOI:** 10.1099/jmm.0.002166

**Published:** 2026-05-18

**Authors:** Rong Chen, Yuanyuan Xu

**Affiliations:** 1 Department of Infectious Diseases, Xinchang County People’s Hospital, Shaoxing City, PR China; 2 Respiratory Medicine Department, Zhuji Affiliated Hospital of Wenzhou Medical University, Shaoxing City, PR China

**Keywords:** MERTK, NLRC4 inflammasome, polarization of macrophages, *Pseudomonas aeruginosa*

## Abstract

**Introduction.** As a leading cause of severe pulmonary infections, such as hospital-acquired pneumonia, *Pseudomonas aeruginosa* (PA) poses a significant threat to public health. Macrophage polarization plays a central role in the control of PA infection; however, its precise regulatory mechanisms remain to be fully elucidated.

**Hypothesis.** It remains unclear whether MERTK participates in the regulation of macrophage polarization induced by PA infection, as well as its potential downstream molecular mechanisms, especially the association with the NLRC4 inflammasome.

**Aim.** This study was designed to investigate the specific role and underlying molecular mechanisms of MERTK-mediated macrophage phenotypic switching during PA infection, with the goal of defining the MERTK–NLRC4 macrophage polarization regulatory axis.

**Methodology.** The expression dynamics of MERTK in alveolar macrophages from PA-infected mice were detected via Reverse transcription quantitative real-time PCR (RT‑qPCR) and Western blotting (WB). Flow cytometry was employed to determine the proportions of M1 (CD86+F4/80+) and M2 (CD206+F4/80+) macrophages. ELISA was utilized to quantify the levels of M1-associated (TNF-*α*, IL-6) and M2-associated [IL-10, transforming growth factor (TGF)-*β*] inflammatory cytokines, while the phagocytic activity of macrophages against PA was detected. WB was further applied to detect the expression of cleaved caspase-1 and N-GSDMD in the NLRC4 inflammasome pathway. The secretion of IL-1*β* and IL-18 and the release of lactate dehydrogenase were assessed.

**Results.** PA infection induced the upregulation of MERTK in alveolar macrophages. MERTK knockdown facilitated macrophage polarization toward the M1 phenotype while suppressing M2 polarization. Mechanistically, MERTK knockdown impaired NLRC4 inflammasome activation. Functional rescue experiments validated that MERTK overexpression activated M2 polarization by activating NLRC4 inflammasomes, which was reversed by NLRC4 knockdown.

**Conclusion.** By upregulating MERTK to activate NLRC4 inflammasomes, PA facilitated M2 polarization of alveolar macrophages. This discovery furnished a critical theoretical foundation for the development of novel therapeutic strategies for PA infection targeting the MERTK–NLRC4 inflammasome–macrophage polarization axis.

## Data Summary

The data and materials in the current study are available from the corresponding author.

## Introduction

*Pseudomonas aeruginosa* (PA) is a multidrug-resistant Gram-negative opportunistic pathogen that extensively exists in the natural environment; PA infection can lead to acute and chronic pneumonia [1]. PA ranks among the primary pathogens responsible for hospital-acquired pneumonia and ventilator-associated pneumonia [2,3]. For patients in intensive care units, particularly those requiring mechanical ventilation support, the onset of PA pneumonia is often associated with rapid clinical deterioration. Pneumonia may progress rapidly to severe necrotizing pneumonia, bacteraemia and sepsis, leading to increased patient mortality [4]. Thus, in-depth investigation into the pathogenic mechanisms underlying the interaction between PA and the host lung, as well as the development of novel therapeutic strategies that can eliminate its pathogenic pathways, holds essential clinical relevance and societal value.

The normal defence responses of the lung encompass innate and adaptive immunity. Innate immune defence involves airway architecture, antimicrobial molecules, alveolar macrophages and neutrophils [5,6]. Within this intricate network, alveolar macrophages, which serve as ‘sentinels’ stationed at the forefront of the airways and alveolar spaces, exert a central role in the initial recognition of PA infection, regulation of immune responses and determination of disease outcomes [7]. Macrophages can polarize into distinct phenotypes in response to microenvironmental signals, and this plasticity is essential during the progression of PA infection [8]. Classically activated M1 macrophages primarily respond to signals such as IFN-*γ*, exhibit potent antibacterial activity and pro-inflammatory properties and serve as the primary effector cells for PA clearance; alternatively activated M2 macrophages respond to signals including IL-4 and IL-13, with core functions in suppressing inflammation, facilitating tissue repair and promoting fibrosis [9]. In acute PA infection, sufficient M1 polarization is essential for effective bacterial clearance; however, immunopathological damage to lung tissues can be exacerbated via excessive or prolonged M1 responses. Conversely, premature or excessive switching to the M2 phenotype may alleviate inflammation and cause incomplete bacterial clearance, thereby driving the chronicity of infection [10,11]. Nevertheless, the dynamic regulatory mechanisms governing macrophage polarization during PA infection remain incompletely elucidated.

The protein encoded by the c-mer tyrosine kinase (MERTK) gene is a pivotal member of the TAM receptor tyrosine kinase family, with high expression in macrophages [12]. Beyond facilitating the clearance of apoptotic debris to maintain tissue homeostasis, activation of MERTK in macrophages also directly sends negative regulatory signals to dampen excessive innate immune responses [13]. MERTK signalling tends to activate macrophage differentiation toward the anti-inflammatory and reparative M2 phenotype while restricting polarization toward the pro-inflammatory and antibacterial M1 phenotype [14]. PA infection induces upregulated MERTK expression in alveolar macrophages, thereby mitigating excessive inflammation and restoring tissue homeostasis [15]. However, the regulatory mechanism by which MERTK modulates inflammatory responses, particularly its role in PA infection-mediated macrophage phenotypic switching, remains unelucidated.

In the present investigation, MERTK expression is upregulated in PA-infected mouse-derived macrophages and functions to facilitate macrophage polarization toward the M2 phenotype. Mechanistically, MERTK activates the NLRC4 inflammasome, thereby mediating the phenotypic switching of alveolar macrophages. Overall, we uncover the role of MERTK-mediated macrophage M2 polarization in PA-infected alveolar macrophages and elucidate the function of the NLRC4 inflammasome in this process, providing potential therapeutic targets and strategies for patients with PA infection.

## Methods

### Cell cultivation

MHS cells (mouse alveolar macrophage cell line, CL-0597) were derived from Wuhan Procell Life Science and Technology Co., Ltd (China). Cells were cultivated in RPMI-1640 medium (Gibco, USA) supplemented with 10% fetal bovine serum, 100 U ml^−1^ penicillin and 100 µg ml^−1^ streptomycin (Gibco) and were maintained in a humidified incubator (37 °C, 5% CO_2_). When cell confluence reached 80–90%, subculture was performed.

### Strain culture and infection

PA strain PA14 (Q2058) was obtained from Hangzhou Baosai Biotechnology Co., Ltd (China). In the context of application, a single colony of PA14 was inoculated into Luria–Bertani (LB) liquid medium and cultured overnight at 37 °C with shaking at 220 r.p.m. An appropriate amount of the overnight culture was transferred to fresh LB medium at a 1:100 ratio and shaken until mid-logarithmic growth phase (OD600≈0.6). The bacteria were collected by centrifugation and resuspended in medium without penicillin and streptomycin. Subsequently, PA14 bacterial suspension was added to the cell culture plate at the m.o.i. of 25 for infection.

### Cell transfection

The MERTK-specific short hairpin RNA (sh-MERTK), the specific NLRC4 knockdown shRNA (sh-NLRC4), the plasmid-overexpressing MERTK (oe-MERTK) and their respective negative controls were all synthesized by GenePharma (China). Transfection was carried out with Lipofectamine 2000 transfection reagent (Thermo Fisher, USA). Transfection efficiency was validated 48 h post-transfection.

### Reverse transcription quantitative real-time PCR (RT‑qPCR)

Macrophages from different treatment groups were harvested, and total RNA was isolated by TRIzol reagent. The concentration and purity of the extracted RNA were quantified via a NanoDrop spectrophotometer (Thermo Fisher). Reverse transcription of RNA into cDNA was conducted using the PrimeScript RT reagent kits (Takara, Japan). Amplification was carried out on an Applied Biosystems 7500 Fast instrument (Thermo Fisher) with the synthetic cDNA as template using the SYBR Premix Ex Taq kit (Takara). The 2^-ΔΔCt^ method was the method for calculating relative gene expression, with GAPDH as the internal reference. The primer sequences are listed in Table 1.

**Table 1.** Primer sequences

Gene name	Primer sequence (5′→3′)
MERTK	Forward primer: AGTTTGGGACGTTGGTGGAT
	Reverse primer: GGACACCGTCAGTCCTTTGT
NLRC4	Forward primer: AATTCAGATGGGCAGACAGG
	Reverse primer: GAGCCCTATTGTCACCAGGA
GAPDH	Forward primer: TGAAGGGTGGAGCCAAAAG
	Reverse primer: AGTCTTCTGGGTGGCAGTGAT

### Western blotting

Macrophages from distinct treatment groups were harvested and lysed in RIPA lysis buffer (Beyotime, China) to extract total protein. Protein concentrations were determined using a bicinchoninic acid protein assay kit (Beyotime). Protein samples were isolated by SDS-PAGE and transferred onto PVDF membranes. Following transfer, the membranes were blocked with 5% non-fat milk for 1 h at room temperature. The blocked membranes were then incubated overnight at 4 °C with specific primary antibodies against the following targets: MERTK (ab300136), NLRC4 (ab201792), pro-caspase-1 (ab179515), cleaved caspase-1 (ab148313), GSDMD (ab209845) and GAPDH (ab9485; all from Abcam, UK). On the subsequent day, the membranes were rinsed three times with Tris-buffered saline with Tween and incubated with the corresponding horseradish peroxidase-conjugated goat anti-rabbit IgG secondary antibody (ab6721, Abcam) for 1 h at room temperature. After extensive washing, signals were developed with an enhanced chemiluminescence detection kit (Beyotime) and captured on a ChemiScope 6200 imaging system (Clinx, China).

### Flow cytometry

To identify the M1 and M2 phenotypes, the expression of their specific surface markers, CD86 (M1) and CD206 (M2), was assessed via flow cytometry (FCM). In brief, macrophages from distinct treatment groups were harvested, resuspended in PBS supplemented with 1% BSA (FCM staining buffer). Cell density was adjusted to ~1×10⁷ cells ml^−1^. A total of 100 µL of the cell suspension was incubated with fluorescein-labelled mouse anti-F4/80-FITC (E-AB-F0995UC, Elabscience, China), anti-CD86-FITC (105025, BioLegend, USA) and anti-CD206-PE/Cy7 (141720, BioLegend), respectively, at 4 °C for 30 min (without lights). After rinsing twice with 2 ml of FCM staining buffer to remove unbound antibodies, cells were resuspended in 200 µL of PBS and analysed by a flow cytometer (Agilent, USA).

### ELISA

Culture supernatants of macrophages from distinct treatment groups were harvested and centrifuged at 1,000 ***g*** for 10 min at 4 °C to eliminate cellular debris. The resulting supernatants were stored until subsequent assays. Cytokine secretion was detected with the following commercial ELISA kits: TNF-*α* (E-EL-M3063), IL-6 (E-EL-M0044), IL-10 (E-EL-M0046), IL-1*β* (E-EL-M0037) and IL-18 (E-EL-M0730) from Elabscience (China) and TGF-*β* (D721150) from Sangon Biotech (China).

### Lactate dehydrogenase detection

The release of lactate dehydrogenase (LDH) in cell culture supernatants was quantified using an LDH assay kit (C0016, Beyotime). In brief, cell culture supernatants from distinct treatment groups were harvested and centrifuged at 400 ***g*** for 5 min at 4 °C to eliminate suspended cells. A total of 120 µL of the supernatant was carefully aspirated and transferred to a fresh 96-well microplate. The LDH working solution was prepared, with 60 µL of the working solution added to each well. The plate was then incubated for 30 min at room temperature in the dark. Subsequently, the absorbance at a wavelength of 490 nm was quantified via a microplate reader.

### Phagocytic activity assay

To evaluate the phagocytic capacity of macrophages against PA14, the following phagocytosis assay was performed. First, PA14 bacterial suspension with the m.o.i. of 25 was added to macrophages cultured in 24-well plates, followed by co-incubation at 37 °C in a 5% CO_2_ incubator for 2 h to permit phagocytosis. Upon completion of incubation, the supernatant was promptly eliminated, and the cells were gently rinsed three times with pre-cooled PBS to completely eliminate non-internalized (cell surface-adhered) bacteria. Subsequently, PBS containing 1% Triton X-100 was added to each well. The cells were lysed at room temperature for 10 min to release intracellularly internalized bacteria. The cell lysate was subjected to serial tenfold dilutions, and appropriate volumes of the dilutions were uniformly spread onto LB agar plates and incubated overnight at 37 °C. On the following day, c.f.u. were counted, and the total amount of internalized bacteria per well was calculated based on the dilution factor, thus quantitatively assessing the phagocytic activity of macrophages.

### Statistical analysis

Statistical analysis and graphing of data were completed with GraphPad Prism 10.2. Data were presented as mean±sd. One-way ANOVA was employed for comparisons among multiple groups; t-test was conducted for pairwise comparisons between groups. Differences were considered statistically significant when *P*<0.05.

## Results

### PA infection induces the upregulation of MERTK expression in alveolar macrophages

**Fig. 1.**
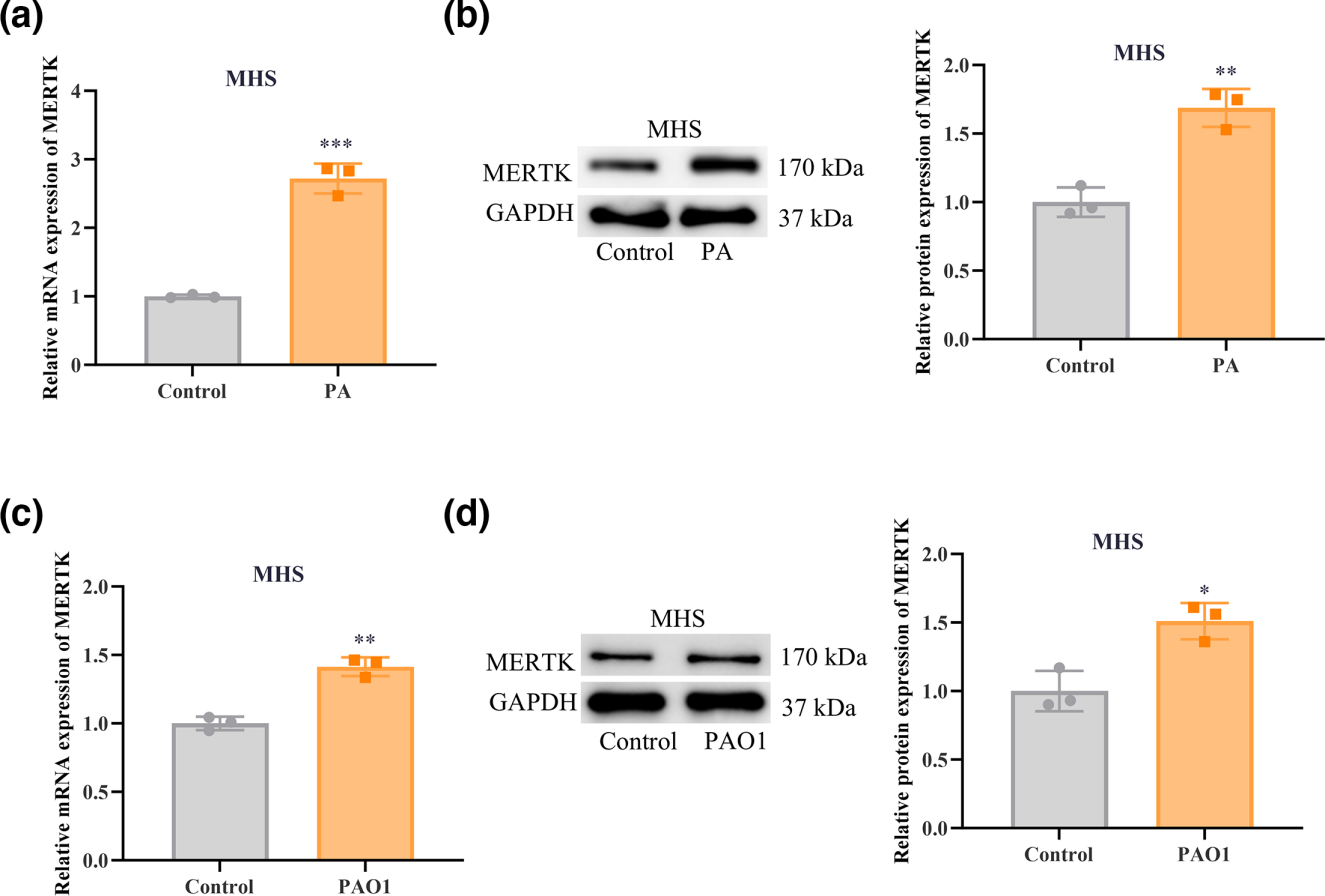
PA infection facilitates MERTK expression in alveolar macrophages. (a) RT‑qPCR is used to detect MERTK mRNA expression in MHS cells infected with PA or uninfected. (b) WB is used to detect the expression of MERTK protein in MHS cells infected with PA or uninfected. (c) Relative mRNA expression of MERTK in MHS cells following PAO1 infection was determined by RT‑qPCR. (d) Relative protein expression of MERTK in MHS cells after PAO1 infection was examined by WB. Data are presented as mean±sd from three independent biological experiments. *P<0.05, **P < 0.01, ***P < 0.001 and ****P < 0.0001.

To investigate the effect of PA infection on MERTK expression, MHS cells were infected with PA14. RT‑qPCR results demonstrated that the MERTK mRNA level in MHS cells from the PA-infected group was markedly elevated compared to the control group (Fig. 1a). Furthermore, the protein expression of MERTK was also upregulated in MHS cells in the PA-infected group (Fig. 1b). To verify whether this phenomenon is conserved across different bacterial strains, we further infected MHS cells with the PAO1 strain. The results demonstrated that PAO1 infection also elicited a marked increase in MERTK expression at both the mRNA and protein levels (Fig. 1c, d), with a kinetic profile consistent with that observed in the PA14-infected group. Subsequently, to determine whether the upregulation of MERTK depends on bacterial viability, heat-killed PA14 was included as a control. Compared with the live bacteria-infected group, treatment with heat-killed PA14 failed to significantly induce MERTK expression at the mRNA level (Fig. S1A available in the online Supplementary Material) or protein level (Fig. S1B), with levels remaining comparable to those of the untreated control group. These findings suggest that the PA-induced upregulation of MERTK is likely dependent on the viability of the bacterium. Collectively, PA infection upregulated MERTK expression in alveolar macrophages.

### Knockdown of MERTK suppresses M2 polarization of alveolar macrophages

**Fig. 2.**
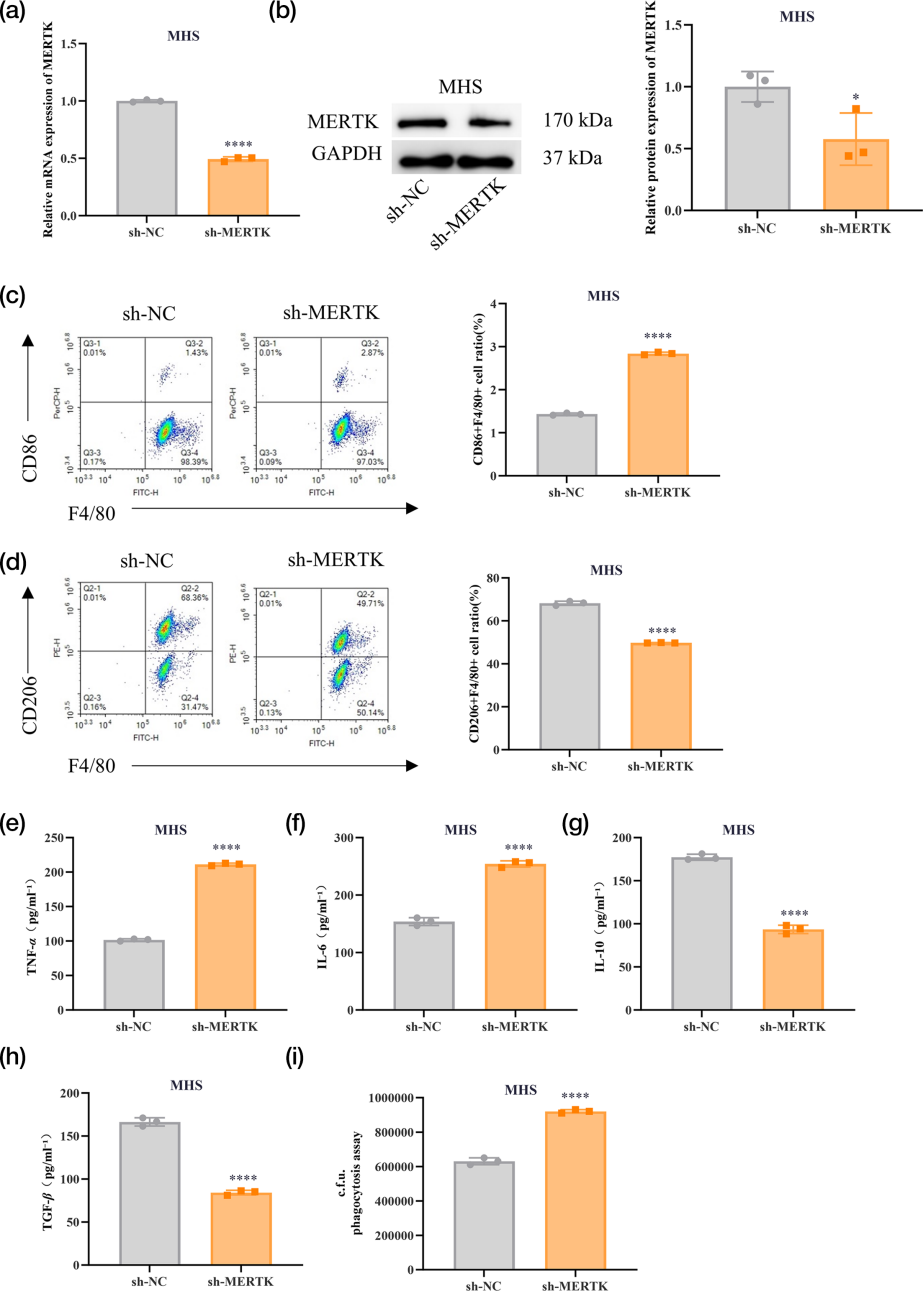
Knockdown of MERTK inhibits M2 polarization of alveolar macrophages. (a, b) MHS cells are treated with PA for 2 h, followed by transfection with sh-MERTK or sh-NC. The transfection efficiency is verified by RT‑qPCR and WB. (c, d) The proportion of CD86+F4/80+and CD206+F4/80+ macrophages is detected by FCM. (e–h) ELISA is applied to detect the levels of M1 macrophage-related cytokines TNF-*α* and IL-6 and M2 macrophage-related cytokines IL-10 and TGF-*β* in the culture supernatant of macrophages. (i) Phagocytosis assays are performed after lysis of the bacteria, and c.f.u. data represents the number of internalized bacteria in a 2-h period. Data are presented as mean±sd from three independent biological experiments. *P<0.05, **P < 0.01, ***P < 0.001 and ****P < 0.0001.

MHS cells infected with PA were transfected with sh-MERTK or sh-NC. RT‑qPCR and Western blotting (WB) analyses demonstrated that MERTK knockdown reduced both MERTK mRNA and protein expression levels in MHS cells, confirming successful transfection (Fig. 2a, b). Subsequently, the polarization status of MHS cells was assessed via FCM. As depicted in Fig. 2(c, d), MERTK knockdown led to an increased proportion of CD86+F4/80+ macrophages and a decreased proportion of CD206+F4/80+ macrophages. In addition, MERTK knockdown elevated the levels of TNF-*α* and IL-6 in MHS cell culture supernatants, while reducing the levels of IL-10 and TGF-*β* (Fig. 2e–h). Concurrently, MERTK knockdown markedly enhanced the phagocytic activity of MHS cells compared to the control group (Fig. 2i). Collectively, MERTK knockdown suppressed M2 polarization of alveolar macrophages.

### MERTK knockdown impairs NLRC4 inflammasome activation

**Fig. 3.**
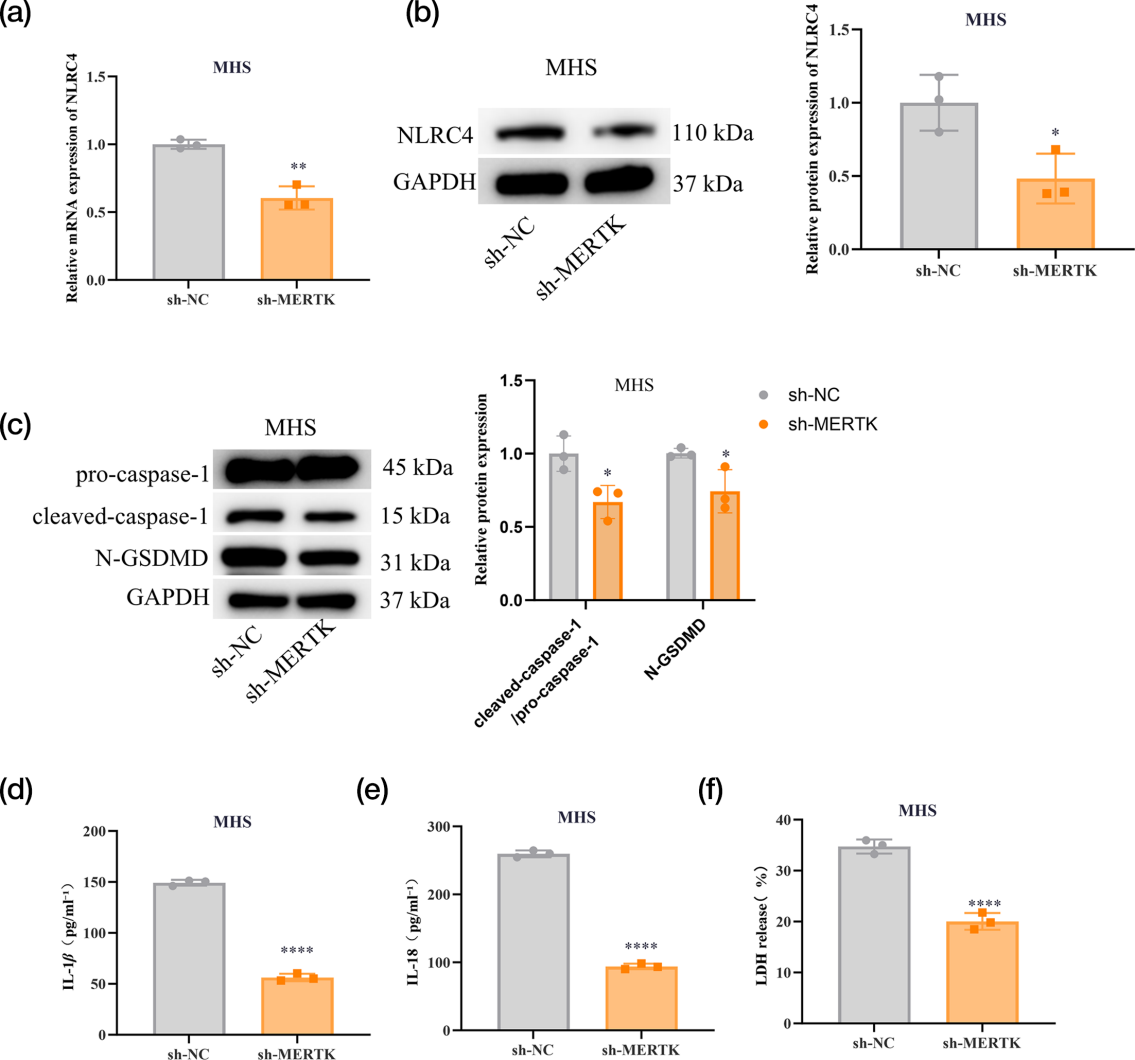
MERTK knockdown impairs NLRC4 inflammasome activation. (a, b) MHS cells are treated with PA for 2 h, followed by transfection with sh-MERTK or sh-NC. The expression of NLRC4 is detected by RT‑qPCR and WB. (c) WB is applied to detect the expression of pro-caspase-1, cleaved caspase-1 and N-GSDMD proteins. (d, e) ELISA is employed to detect the secretion of IL-1*β* and IL-18 in cell culture supernatant. (f) Kits are used to detect LDH release. Data are presented as mean±sd from three independent biological experiments. *P<0.05, **P < 0.01, ***P < 0.001 and ****P < 0.0001.

To investigate the effect of MERTK on the NLRC4 inflammasome, RT‑qPCR and WB analyses were performed. Both NLRC4 mRNA and protein expression levels in MHS cells were markedly downregulated via MERTK knockdown (Fig. 3a, b). In addition, compared to the control group, the protein expression of cleaved caspase-1 and N-GSDMD in MHS cells was reduced by MERTK knockdown (Fig. 3c). The secretion of IL-1*β* and IL-18 in cell culture supernatants was measured using ELISA; decreased concentrations of both cytokines were observed following MERTK knockdown (Fig. 3d, e). MERTK knockdown also reduced LDH release (Fig. 3f). Collectively, NLRC4-dependent inflammasome activation was suppressed by MERTK knockdown.

### MERTK activation of the NLRC4 inflammasome facilitates M2 polarization in macrophages

**Fig. 4.**
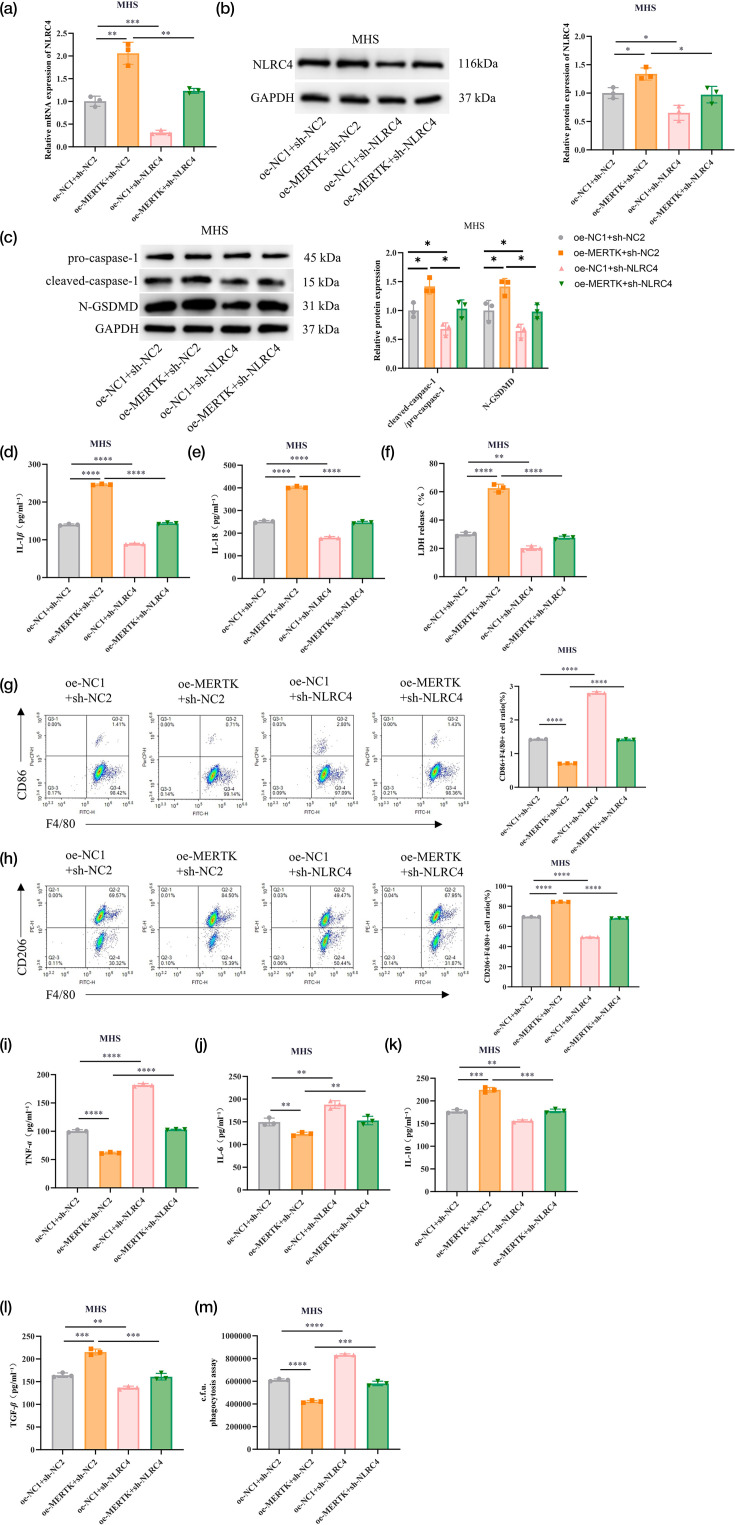
MERTK activation of the NLRC4 inflammasome facilitates M2 polarization of macrophages. (a, b) MHS cells are treated with PA for 2 h and transfected with oe-MERTK and sh-NLRC4, while the corresponding negative controls were set. NLRC4 expression is detected by RT‑qPCR and WB. (c) WB is employed to detect the expression of pro-caspase-1, cleaved caspase-1 and N-GSDMD proteins. (d, e) ELISA is employed to detect the secretion of IL-1*β* and IL-18 in cell culture supernatant. (f) Kits are applied to detect LDH release. (g, h) The proportion of CD86+F4/80+ and CD206+F4/80+ macrophages is determined by FCM. (i–l) ELISA is employed to detect the levels of M1 macrophage-related cytokines TNF-*α* and IL-6 and M2 macrophage-related cytokines IL-10 and TGF-*β* in the culture supernatant of macrophages. (m) Phagocytosis assays are performed after lysis of the bacteria, and c.f.u. data represents the number of internalized bacteria in a 2-h period. Data are presented as mean±sd from three independent biological experiments. *P<0.05, **P < 0.01, ***P < 0.001 and ****P < 0.0001.

To investigate whether M2 polarization in macrophages was facilitated by MERTK activation of the NLRC4 inflammasome, rescue experimental groups were established in MHS cells, including MERTK overexpression and NLRC4 knockdown. RT‑qPCR and WB analyses demonstrated that NLRC4 mRNA and protein expression in MHS cells were markedly upregulated by MERTK overexpression, while the effects of MERTK overexpression were attenuated by NLRC4 knockdown (Fig. 4a, b). Furthermore, the detection of key NLRC4 inflammasome proteins revealed that increases in cleaved caspase-1 and N-GSDMD protein levels induced by MERTK overexpression were reversed by NLRC4 knockdown (Fig. 4c). In addition, the enhanced secretion of IL-1*β* and IL-18 and LDH release by MERTK overexpression were reversed by NLRC4 knockdown (Fig. 4d–f). FCM analysis demonstrated that the proportion of CD86+F4/80+ macrophages was reduced and that of CD206+F4/80+ macrophages was increased by MERTK overexpression; however, these phenotypic changes were reversed upon NLRC4 knockdown (Fig. 4g, h). Meanwhile, MERTK overexpression reduced TNF-*α* and IL-6 secretion, increased IL-10 and TGF-*β* level in MHS cell supernatants and suppressed MHS cell phagocytosis; these effects were reversed by NLRC4 knockdown (Fig. 4i–m). Collectively, MERTK facilitated M2 macrophage polarization via activation of the NLRC4 inflammasome.

## Discussion

As sentinel cells of the innate immune system, macrophages exert pivotal roles in immune defence against infections, maintenance of tissue homeostasis and host protection via phagocytosis [16]. We investigated the specific function and underlying molecular mechanism of MERTK-mediated macrophage phenotypic transition during PA infection and delineated the PA–MERTK–NLRC4 inflammasome–macrophage polarization regulatory axis.

During PA infection, highly virulent strains enhanced their environmental adaptability and antimicrobial resistance by upregulating genes associated with nutrient acquisition and antimicrobial efflux, thereby driving the chronicity of infection [17]. Correspondingly, alveolar macrophages within the host activated an adaptive immune regulatory network to maintain inflammatory homeostasis in response to bacterial invasion [18,19]. MERTK expression was found to be upregulated at both the transcriptional and protein levels in alveolar macrophages of PA-infected mice, implying that MERTK, as a critical immunomodulatory molecule, was likely implicated in the host response to PA infection. The core functions of MERTK have been well-documented, including mediating the clearance of apoptotic cells and negatively regulating innate immune responses; its upregulation is typically regarded as a hallmark of the body initiating anti-inflammatory and tissue repair programmes [20,21]. Notably, accumulating evidence has highlighted the essential role of MERTK in regulating macrophage polarization. For instance, in a sepsis-associated acute kidney injury model, ADAM17 was found to promote macrophage polarization toward the M2 phenotype by upregulating MERTK expression, ultimately alleviating renal tubular epithelial cell apoptosis and inflammatory responses [22]. In addition, Chen *et al*. [14] reported that MERTK induced M2 macrophage polarization via activation of the PI3K/Akt/GSK-3*β* signalling pathway, thereby exerting anti-inflammatory effects in a gout inflammation model. The critical role of MERTK in macrophage polarization in the context of PA infection was validated in this study. Experimental results clearly demonstrated that MERTK knockdown facilitated macrophage polarization toward the pro-inflammatory M1 phenotype while inhibiting their transition to the anti-inflammatory, reparative M2 phenotype. MERTK is a pivotal gene regulating macrophage polarization during PA infection, underscoring its potential as a therapeutic target.

The NLRC4 inflammasome, as a multiprotein complex in the innate immune system, can be activated by specific components of a certain pathogen, such as flagellin or the type III secretion system of PA [23]. NLRC4 inflammasome’s canonical function involves recruiting and activating caspase-1, which drives the maturation and secretion of pro-inflammatory cytokines IL-1*β* and IL-18, thereby triggering pyroptosis, critical for host defence against intracellular bacteria and certain Gram-negative pathogens, including PA [24]. NLRC4 activity is closely linked to the regulation of immune responses, including macrophage polarization. For example, a study in a mouse model of non-alcoholic fatty liver disease exhibited that NLRC4 knockdown reduced M2 macrophage polarization [25]. Thus, whether NLRC4 modulates M2 polarization of macrophages during PA infection was further explored in this study. Upregulated MERTK expression in PA-infected alveolar macrophages critically promoted NLRC4 inflammasome activation. Rescue experiments exhibited that MERTK overexpression facilitated M2 macrophage polarization via activating the NLRC4 inflammasome, while subsequent NLRC4 knockdown reversed the effect of MERTK overexpression. The MERTK–NLRC4 inflammasome axis is a pivotal regulatory pathway directing macrophage polarization during PA infection. However, this study still has the following limitations: First, although the MHS cell line, as an SV40-immortalized mouse alveolar macrophage cell line, possesses advantages such as stable cell source and good reproducibility in *in vitro* studies, it may differ from primary alveolar macrophages in certain biological characteristics. Therefore, the MERTK–NLRC4 regulatory axis observed in the MHS cell line in this study requires further validation in primary alveolar macrophages or *in vivo* animal models. In future research, we will prioritize the isolation of primary cells or the establishment of a PA-infected mouse model to confirm the authenticity and importance of this regulatory pathway under physiological conditions. Second, while this study mainly focused on the effect of the MERTK–NLRC4 axis on macrophage M2 polarization, the upstream signalling molecules responsible for the upregulation of MERTK during PA infection and their specific bacterial virulence factors remain incompletely understood and require further clarification.

In conclusion, MERTK was highly expressed in PA-infected alveolar macrophages, and its knockdown inhibited M2 macrophage polarization. The NLRC4 inflammasome was involved in MERTK-mediated M2 polarization of macrophages during PA infection. A MERTK–NLRC4 inflammasome–macrophage polarization signalling cascade was established. This enriches the molecular pathogenesis theory of PA infection and provides experimental evidence for the development of anti-infective therapeutics targeting MERTK or NLRC4.

## Supplementary material

10.1099/jmm.0.002166 Uncited Fig. S1.

## Abbreviations

FCM: flow cytometry
LB: Luria–Bertani
LDH: lactate dehydrogenase
PA: *Pseudomonas aeruginosa*
RT‑qPCR: reverse transcription quantitative real-time PCR
TGF: transforming growth factor
WB: Western blotting
