# Supplementary material for: Pseudomonas aeruginosa infection is associated with alveolar macrophage M2 polarization via MERTK-mediated NLRC4 inflammasome activation
Source: J Med Microbiol. 2026 May 18;75(5):002166. doi: 10.1099/jmm.0.002166 (PMC13183294; doi:10.1099/jmm.0.002166)
Supplement: Uncited Fig. S1. [file jmm-75-02166-s001.pdf]

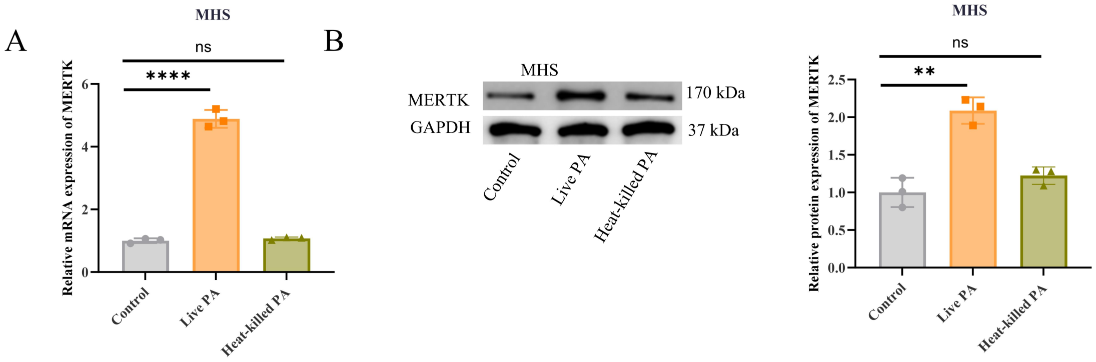

Figure S1. Upregulation of MERTK is Associated with the Viability of PA PA14

A: mRNA expression levels of MERTK in MHS cells treated with heat-killed PA14 were determined by qPCR. B: Changes in MERTK protein levels and quantitative analysis in MHS cells following treatment with heat-killed PA14 were examined by WB. Data are presented as mean ± SD from three independent biological experiments. * indicates $P < 0.05$, $**P < 0.01$, $***P < 0.001$, $****P < 0.0001$.
